# The Therapeutic Potential of Glymphatic System Activity to Reduce the Pathogenic Accumulation of Cytotoxic Proteins in Alzheimer’s Disease

**DOI:** 10.3390/ijms26157552

**Published:** 2025-08-05

**Authors:** Kamila Kopeć, Dariusz Koziorowski, Stanisław Szlufik

**Affiliations:** Department of Neurology, Faculty of Health Sciences, Medical University of Warsaw, Żwirki i Wigury 61, 02-091 Warsaw, Poland

**Keywords:** Alzheimer’s disease, glymphatic system, amyloid-β oligomers, neurodegeneration, aquaporin-4, neuroinflammation

## Abstract

Neurodegenerative disorders, including Alzheimer’s disease (AD), are a growing problem in aging society. The amyloid cascade hypothesis has recently been questioned, and therapies based on it have not yielded the expected results. However, the role of amyloid-β (Aβ) in AD pathogenesis cannot be rejected. It appears that some of the key players in the pathogenesis of the disease are the soluble amyloid-β oligomers. Soluble amyloid-β oligomers have neurotoxic effects by disrupting intracellular Ca^2+^ homeostasis and impairing mitochondrial function. The glymphatic system is an important pathway for the removal of soluble amyloid forms from the brain. The decline in the activity of this system is observed in aging brains, which is correlated with the occurrence of Alzheimer’s disease, primarily among the elderly population. Therefore, the question arises as to whether the glymphatic system could be another potential target for therapeutic interventions in Alzheimer’s disease. In this regard, it is imperative to pay attention to the factors that contribute to the pathogenesis of Alzheimer’s disease and also impact the glymphatic system, such as sleep, physical activity, alcohol consumption, and supplementation with polyunsaturated fatty acids. The question remains whether the glymphatic system will become the key to treating Alzheimer’s disease.

## 1. Background

Alzheimer’s disease (AD) is the most prevalent neurodegenerative disease and a common cause of dementia in the elderly population [1]. Progressive impairment of cognitive function is observed [2]. Numerous hypotheses have been proposed regarding the pathogenesis of this disease; however, at present, it is not feasible to define it unambiguously [2]. The abnormal accumulation of misfolded proteins is often mentioned as a major factor in the pathogenesis of AD, and the presence of protein deposits in the brain is considered a pathological hallmark of AD [3]. Nevertheless, it appears that the pathogenesis of AD is intricate, with numerous interdependent components influencing its pathogenesis [2,3]. The median survival time following a diagnosis of AD is 8.3 years for patients diagnosed with AD at the age of 65 years and 3.4 years for patients diagnosed with AD at the age of 90 years. This is associated with an approximately 67% reduction in the median life span of patients diagnosed in their 60s and a 39% reduction in the median life span of patients diagnosed in their 90s [4]. Moreover, the quality of life of patients gradually deteriorates over the course of the disease; they begin to lose independence and require constant care, which affects the patient’s immediate environment and their caregivers [5,6]. The incidence of AD is strongly associated with increasing age, which may have particular consequences given the increasing rate of population aging [6]. In the United States alone, an estimated 6.7 million people suffer from AD. However, if there are no breakthroughs in treating or slowing this disease in the near future, this number could increase to an estimated 13.8 million by 2060 [7]. In the United States, the total payment for healthcare for people with dementia in 2023 is estimated to have been USD 345 billion. Moreover, according to data from 2021, AD is the seventh leading cause of death [7]. In Europe, the prevalence of AD was estimated at 5.05%, with rates of 3.31% in men and 7.13% in women, both increasing with age [8]. Effective treatment or prevention of AD constitutes one of the greatest challenges in modern neurology.

## 2. The (Un)certain Role of Amyloid-β in Neurodegeneration

The plaques and neurofibrillary tangles identified in the brain histology of patients studied by Alois Alzheimer have become a significant focus of research concerning the pathogenesis of Alzheimer’s disease (AD) [9,10,11]. A common feature of neurodegenerative diseases is the aggregation of misfolded proteins, which contribute to the pathological process of neurodegeneration [12]. In AD, the formation of plaques containing amyloid-β (Aβ) and neurofibrillary tangles composed of hyperphosphorylated tau is observed [13]. This raises the question of the underlying processes that lead to the accumulation of these proteins and their impact on the central nervous system.

β-amyloid precursor protein (APP) is a transmembrane protein expressed at high levels in the brain [14]. APP and APP-like proteins (APLPs) play important physiological roles in the central and peripheral nervous systems. Their range of functions includes nervous system development, synaptogenesis, and synaptic functions, including synaptic plasticity, learning, and memory, as well as axonal growth and guidance [15]. Importantly, this molecule is characterized by a complex metabolic pattern. Its metabolism, depending on the APP-cleaving enzymes involved in the process, can proceed along amyloidogenic and non-amyloidogenic pathways [16]. The non-amyloidogenic pathway involves α- and γ-secretases, and their products are a long-secreted form of APP (sAPPα) and C-terminal fragments. However, the amyloidogenic pathway involving β- and γ-secretases leads to the cleavage of APP into its long secreted form (sAPPβ), C-terminal fragments, and Aβ [16]. Aβ is a peptide whose monomers undergo oligomerization and assemble into highly heterogeneous oligomeric complexes. In the next stage, soluble Aβ oligomers aggregate into insoluble beta sheets, which form amyloid fibrils and plaques [17]. Hardy and Higgins posited that the accumulation of Aβ in the brain parenchyma was the fundamental cause of AD pathogenesis more than two decades ago. They then formulated the amyloid cascade hypothesis, which was as follows: “*Our hypothesis is that deposition of amyloid β protein (AβP), the main component of the plaques, is the causative agent of Alzheimer’s pathology and that the neurofibrillary tangles, cell loss, vascular damage, and dementia follow as a direct result of this deposition*” [18]. This became the starting point of many studies that focused on plaques as the main culprits in the disease process and made them the target of potential therapies [19,20,21]. The amyloid cascade hypothesis is currently under investigation. A discussion of the validity of challenging this hypothesis is beyond the scope of this review and has been described in many articles [22,23,24]. However, some key aspects need to be considered. Unfortunately, anti-amyloid therapies, the cornerstone of which is the amyloid cascade hypothesis, have not produced results commensurate with the high expectations associated with them [25,26,27]. An example of such a therapy is aducanumab, which has been approved by the FDA. Aducanumab is an anti-Aβ monoclonal antibody that is selective towards Aβ aggregates and remains controversial [28]. The phase III trials for aducanumab, EMERGE and ENGAGE, had limited and divergent outcomes with identical regimens. In the ENGAGE group, a 60% reduction in the Aβ burden in the brain was observed, but without a significant improvement in cognitive functions. EMERGE observed a reduction in Aβ load and a relative 22% reduction in cognitive decline [29]. In this case, the reduction in Aβ plaques seems to be associated with a modest improvement in cognition, and this conclusion is uncertain due to divergent research results [29]. Other anti-amyloid molecules that are approved by the FDA include Donanemab and Lecanemab [30]. Donanemab is a monoclonal antibody that primarily targets insoluble forms of amyloid plaques, thereby facilitating their removal [31]. In contrast, Lecanemab also targets soluble Aβ aggregates (oligomers and protofibrils), thereby preventing further Aβ deposition [32]. Research has demonstrated that Donanemab and Lecanemab are effective in reducing amyloid levels in the brain and moderately slowing down cognitive decline [33,34]. The efficacy of these drugs, as measured by scales that indicate slowed cognitive decline, is the subject of attention, as it is the question of whether it translates into clinically meaningful improvements that are perceived by both patients and physicians [35]. Additionally, there are certain concerns regarding the safety profiles of these monoclonal antibodies [35,36]. The anti-Aβ therapies discussed may be associated with the occurrence of amyloid-related imaging abnormalities (ARIAs), which include edema (ARIA-E) and hemorrhage (ARIA-H) [37,38,39]. ARIA events are mostly mild to moderate and often clinically silent, but importantly, it appears that anti-Aβ therapies and the resulting ARIAs may contribute to accelerated brain atrophy, thereby impairing long-term brain health [36]. Certainly, additional research is necessary to address this matter.

There are arguments that contrary to the main assumption about the amyloid cascade, the formation of plaques does not drive pathogenesis but has a protective role. Some studies indicate that especially among the older population, Aβ aggregation is not associated with cognitive decline but is associated with a reduced risk of dementia [40,41,42]. There is a significant discrepancy between a clinical diagnosis of AD and the occurrence of characteristic neuropathological changes. In a prospective study, patients were clinically assessed for dementia, and the prevalence of clinically diagnosed AD was 16%. However, it is noteworthy that in autopsies, the prevalence of neuropathological changes in AD was 33% [43]. Similar conclusions were drawn from a study that indicated that the presence of the APOE ε2 allele (APOE2) in patients is associated with an increased incidence of AD neuropathology but, importantly, a reduced risk of dementia [44]. The pathological role of amyloid plaques is questioned in light of emerging arguments, including the modest results of anti-amyloid therapies. However, the role of Aβ in the pathogenesis of AD cannot be completely rejected. It appears that one of the key players in the pathogenesis of AD, whose role has been underestimated, is soluble Aβ oligomers (AβOs) [45].

**Figure 1:**
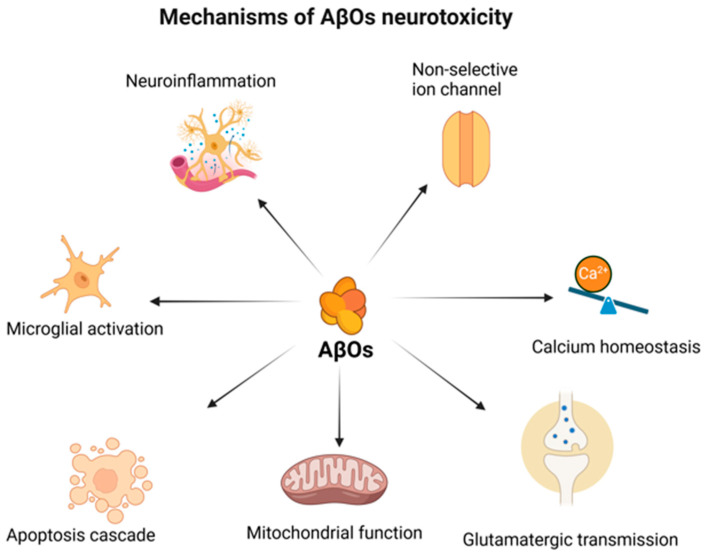
The multidirectional mechanism of AβO toxicity. “Created in BioRender. Kopeć, K. (2025) https://BioRender.com/3uykp0b (accessed on 7 July 2025)”.

Aβ oligomers are highly neurotoxic and can impair hippocampal synaptic plasticity and memory, ultimately leading to cognitive impairment [46]. These molecules exert toxicity through many different mechanisms and induce synaptic loss in AD [17,47,48]. One of the proposed mechanisms of direct neurotoxicity is the formation of voltage-independent, non-selective ion channel pores in the neuronal membranes by AβOs [49]. This in turn results in the disruption of intracellular Ca^2+^ homeostasis, which is another driver of neurodegeneration in AD [50]. Increased levels of cytosolic Ca^2+^ can cause synapse loss and axon atrophy [51]. Increased levels of Ca^2+^ activate calpains. Calpains are a family of Ca^2+^-dependent cysteine proteases that cleave signal transduction proteins and synaptic proteins, including N-methyl-D-aspartic acid (NMDA) glutamate receptors and metabotropic glutamate receptor (GluR1), which are involved in the processes of learning and memory [51,52]. Disturbed calcium homeostasis is also associated with the impairment of glutamatergic synaptic transmission, a characteristic of AD [53]. Although glutamate is one of the key excitatory transmitters in the brain, it can become a potent neurotoxin in non-physiological conditions [54]. Activation of the NMDA receptors is one of the major causes of glutamate toxicity. The activation of NMDA receptors results in Ca^2+^-dependent cell death. The prolonged overload of Ca^2+^ results in the impairment of synaptic function, which is subsequently followed by synaptotoxic effects and ultimately cell death [55]. The abnormal release of glutamate in the hippocampal neurons is a mechanism through which AβOs promote the extracellular accumulation of glutamate [56]. It is noteworthy that some studies have also demonstrated that AβOs play a significant role in the impaired recycling of glutamate in the synapses through the inhibition of glutamate uptake, which in turn may significantly enhance its extracellular concentrations [57]. A study conducted on hippocampal slices revealed that AβOs involved in the activation of specific NMDA receptors inhibit long-term potentiation (LTP), which is one of the mechanisms facilitating learning and memory storage in neuronal circuits [58,59]. Another mechanism involves the impairment of mitochondrial function by AβOs, which results in the disruption of cellular bioenergetics. Studies indicate that AβOs can induce mitochondrial oxidative stress and mitochondrial DNA (mtDNA) damage [60]. Studies have also shown that AβOs disrupt mitochondrial transport and integrity, which may negatively affect synaptic function [61]. It has been demonstrated that AβOs can initiate neuronal cell death by triggering the apoptosis cascade through the release of cytochrome C from the mitochondria. What is particularly intriguing is that this is possible even in the absence of a death signal. AβOs possess the ability to hijack the apoptotic pathway, thereby triggering the formation of apoptotic BAK pores and cytochrome C release independently of apoptotic signals [62]. When mentioning the influence of AβOs on the key elements of AD pathogenesis, it is impossible to ignore neuroinflammation, which is one of the cornerstones of neurodegeneration [63]. The microglia, a specialized population of macrophage-like cells, are responsible for the fundamental functions of CNS homeostasis, including the release of neurotrophic factors, cytokines, and chemokines in response to infection or cell injury, thereby restoring homeostasis [64,65]. In AD, the microglia are in a pathological state of chronic activation, producing pro-inflammatory cytokines, which results in a persistent state of neuroinflammation and consequently neurodegeneration [66,67]. It must be pointed out that there is a correlation between AβOs and microglial activation [68]. In the literature, the significance of AβOs as inducers of the microglial pro-inflammatory phenotype is emphasized, and it is noteworthy that they exert a more potent effect than Aβ fibrils [69,70]. Furthermore, in an in vitro study, it was demonstrated that AβO-stimulated microglia, through the activation of TNF-α signaling, could induce necroptosis and extensive neurodegeneration [71]. AβOs promote the processing of pro-inflammatory IL-1β into a mature form in the microglia. This occurs via mitochondrial reactive oxygen species (ROS)-dependent activation of the NLRP3 inflammasome, which in turn enhances microglial neurotoxicity [72]. AβOs can also directly interact with the NLRP3 inflammasome, which is a protein complex triggering pro-inflammatory cytokine secretion. Interestingly, it was shown that only AβOs, but not monomers or fibrils, were able to activate the intracellular NLRP3 inflammasome, leading to IL-1β secretion [73]. It is particularly interesting that APOE-ε4, which is acknowledged as one of the most significant genetic risk factors for the development of AD, has been demonstrated to stabilize Aβ in its oligomeric form, thereby blocking further aggregation of AβOs into less toxic forms [74,75]. There are numerous mechanisms through which AβOs exert neurotoxic effects (Figure 1). Considering these examples, it appears that it is important to pursue further research on the efficacy of therapies aimed at AβOs. In this regard, it is worth discussing the glymphatic system and its potential impact on AD pathogenesis.

When discussing the role of AβOs in the pathogenesis of AD, it is important to highlight the related role of tau protein. Tau is a protein that plays a significant role in stabilizing the neuronal microtubules [76]. Under physiological conditions, it is soluble and shows a minimal tendency to aggregate [77]. In pathological conditions, such as AD, tau becomes neurotoxic and can aggregate into neurofibrillary tangles [77]. Tau protein and Aβ pathology are linked [78,79]. AβOs can induce neurotoxicity by promoting the hyperphosphorylation of tau through the activation of various kinases, thereby converting tau from its normal functional state into a neurotoxic, aggregation-prone form [78,80]. Conversely, tau is essential for Aβ-induced neurotoxicity [78,81]. A reduction in tau has been shown to ameliorate Aβ-related neurotoxicity [82]. Additionally, soluble toxic aggregates of both Aβ and tau can self-propagate, spreading throughout the brain in a manner similar to prions [78,83,84]. These two pathologies influence each other, creating a vicious cycle that drives AD pathogenesis [78].

## 3. The Glymphatic System—Its Role in Pathogenesis of AD

**Figure 2:**
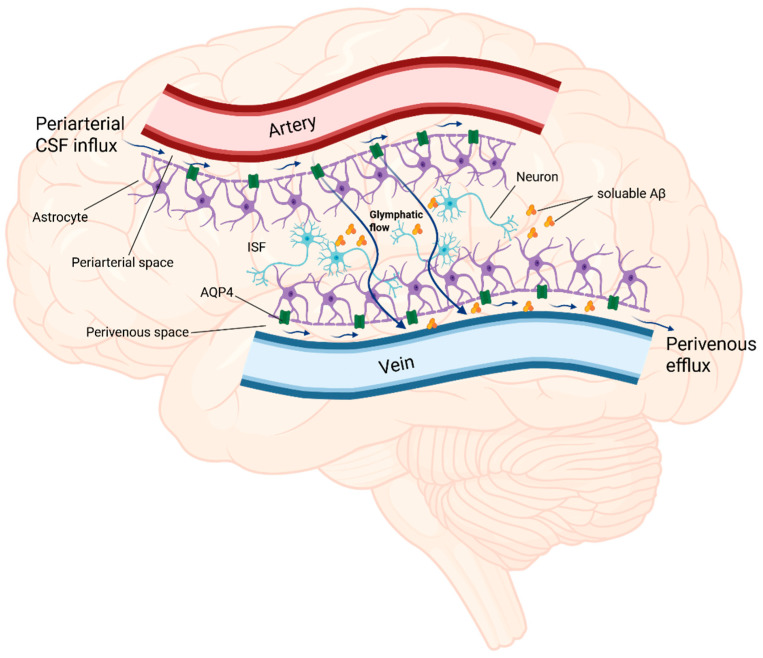
A schematic representation of the glymphatic system. “Created in BioRender. Kopeć, K. (2025) https://BioRender.com/ilhkcp2 (accessed on 7 July 2025)”.

The concept of a glymphatic system is relatively new. The glymphatic system, whose name refers to the glial cells which are vital to the functioning of this system, functions in a similar way to the lymphatic system, as was first described in rodents in 2012 [85]. Nonetheless, numerous studies have confirmed its presence in human brains [86,87,88,89]. The glymphatic system is a complex network of fluids comprising cerebrospinal fluid (CSF) and interstitial fluid (ISF), whose convective flow through the perivascular spaces and brain parenchyma ensures the elimination of soluble proteins and metabolic products from the central nervous system [90]. The glymphatic flow should be considered as a step-by-step process to explain the mechanism of action of the glymphatic system. The initial stage involves the influx of CSF from the subarachnoid space into the perivascular space of the large leptomeningeal arteries. Subsequently, as the vascular tree branches, CSF flows into the perivascular spaces of the penetrating arteries, referred to as Virchow–Robins spaces [85,90,91]. In the second stage, CSF enters the interstitial space of the brain parenchyma and flows through it by mixing with ISF and solutes, which is mediated by astrocytes and aquaporin-4 (AQP4) [85,90]. It is important to emphasize the role of AQP4, which is a water channel on which the glymphatic flow largely depends [92]. For proper functioning of the glymphatic system, the expression pattern and distribution are highly polarized to the perivascular astrocytic end-feet membranes [92]. The end-feet membranes of the astrocytes surround the vessels, and the AQP4 contained within them serves as the entry point for the influx of CSF into the brain parenchyma [90]. Ultimately, in the third stage, the resulting fluid flows through the perivenous spaces and effluxes from the brain via the cervical and meningeal lymphatic vessels (Figure 2) [85,88,90,93]. This directed flow of fluids through the brain contributes to maintaining its homeostasis by washing out metabolic waste with toxic potential [94]. Studies on animal models have indicated an association between impaired function of the glymphatic system and a wide range of neurological disorders, including neurodegenerative diseases such as AD and Parkinson’s disease (PD), stroke, traumatic brain injury (TBI), epilepsy, and autoimmune diseases [91,95,96]. Given the scope of this review, the most important issue in this context is the involvement of the glymphatic system in the pathogenesis and development of AD.

The previous section briefly discussed the role of soluble and insoluble forms of Aβ, identifying the soluble forms as potential crucial players in the pathogenesis of AD. This seems consistent with the currently investigated role of the glymphatic system in the pathogenesis of AD, particularly in soluble Aβ clearance. A study published in 2012, in which the concept of the glymphatic system was used for the first time, reported that fluorescent-tagged soluble Aβ1–40 injected into the brain parenchyma in mice was removed from the brain via the glymphatic pathway. Moreover, this study showed that the disruption of this pathway in AQP4-null mice resulted in a ~55% reduction in Aβ1–40 clearance compared to that in wild-type controls [85]. This study also highlighted the fact that not only can interstitial Aβ be transported by the glymphatic flow but the portion of Aβ dissolved in the CSF can also be transported using the glymphatic pathway [85]. A similar study was conducted in a mouse model of AD with AQP4 knockout, which is crucial to glymphatic flow. It was determined that AQP4 knockout, due to reduced parenchymal clearance of Aβ, leads to the exacerbation of soluble Aβ accumulation and increases the Aβ plaque burden in the brain. Moreover, AQP4 deficiency aggravates cognitive dysfunction in knockout animals [97].

The significance of the glymphatic system in eliminating amyloid from the human brain has been substantiated by research conducted using amyloid positron emission tomography (PET), magnetic resonance imaging (MRI), and neuropsychological evaluations. It was demonstrated that the index of diffusivity along the perivascular space (ALPS index), which is calculated based on MRI and represents the activity of the glymphatic system, exhibited significant negative correlations with the amyloid and tau burden, whereas a positive correlation was observed with cognitive scores in AD patients [98]. It follows that in direct studies on humans, there are indications that glymphatic system activity is correlated with amyloid and tau protein deposition in the brain and cognitive dysfunction [98]. The glymphatic pathway is crucial for Aβ clearance; however, glymphatic clearance and amyloid pathology are interdependent. Impairment of the glymphatic system may be associated with decreased Aβ clearance, but amyloid itself may also negatively affect glymphatic flow. A reduced Aβ42 concentration in the CSF is a positive biomarker of AD, reflecting the accumulation of Aβ in the brain [99]. In a study using MRI, conclusions were drawn about the activity of the glymphatic system in patients with AD based on various measurements, including the perivascular space volume fraction (PVSVF) and the ALPS index. It has been shown that AD patients had significantly lower Aβ42 in the CSF, and probable impairment of the glymphatic system’s function in patients with AD, expressed as a significantly lower ALPS index and significantly enlarged PVSV compared to those in healthy controls, may be related to Aβ deposition in the brain [100]. The levels of soluble forms of Aβ measured in the CSF and the ISF fluctuate in the brain during the sleep–wake cycle in both animals and humans. During wakefulness, Aβ levels increase, and they decrease during sleep [101,102,103,104]. This may be due to changes in the intensity of glymphatic clearance, which decreases significantly during wakefulness, and changes in the metabolic burden, which, contrary to clearance, increases during wakefulness. It is noteworthy that the magnitude of diurnal Aβ fluctuations decreases in tandem with the sequestration of soluble forms into insoluble plaques that are inaccessible for glymphatic clearance [103,104]. A study investigating the role of genetic variants of AQP4 in the relationship between sleep and Aβ burden in older adults led to some interesting conclusions [105]. It was found that genetic variations in AQP4 may affect sleep quality, as measured by the Pittsburgh Sleep Quality Index total score. Additionally, genetic variations in AQP4 appear to moderate the link between sleep and the Aβ burden in the brain, indicating a modulatory role of AQP4 in the sleep–Aβ relationship [105]. Another study indicated that genetic variation in AQP4 was linked to the accumulation of Aβ, the risk of conversion from a mild cognitive impairment into AD, and cognitive decline [106]. This study also highlighted that the genetic variation in AQP4 might correspond to the function of the glymphatic system, which is involved in the clearance of Aβ [106]. Further studies are required to investigate this undoubtedly interesting issue of the link between genetic variation in AQP4, sleep, and glymphatic clearance of Aβ in the context of AD pathogenesis. It should be noted that in addition to the accumulation of Aβ in the parenchymal plaques, it is also possible for Aβ to accumulate in the vessel walls, which is known as cerebral amyloid angiopathy (CAA). CAA might block the perivascular pathways, leading to impaired glymphatic clearance, which in turn is associated with increased Aβ deposition [107,108]. The glymphatic system also participates in tau protein clearance. In a mouse model of tauopathy, glymphatic clearance was shown to be impaired, as was AQP4 polarization, both of which were correlated with increased tau protein deposition [109]. Furthermore, interventions aiming to pharmacologically inhibit AQP4 function resulted in impaired glymphatic CSF-ISF exchange and thus reduced tau protein clearance. Notably, AQP4 polarization was also found to be impaired in brain regions affected by tauopathy [109]. A similar conclusion was reached in another animal model study [110]. In this research, fluorescence-labeled tau was cleared from the brains of animals through the glymphatic clearance pathway. However, in AQP4 KO mice, both the diffusion process and tau clearance were significantly impaired compared to those in wild-type mice [110]. AQP4 deficiency also aggravated tau deposition and neurodegeneration [110].

Studies report that glymphatic activity decreases with age [111,112]. This correlates with the incidence of neurodegenerative diseases, including AD, which is particularly high in the elderly population. The numbers are overwhelming: the latest Alzheimer’s Association Report shows that 5% of people aged 65 to 74, 13.2% of people aged 75 to 84, and 33.4% of people aged 85 or older have AD [7]. The reasons for the weakening of the glymphatic flow with age include a decrease in AQP4 polarization in the aging brain, which has been confirmed in postmortem studies of human brain tissue [113]. A study conducted in an animal model revealed that changes in AQP4 expression in elderly mice, resulting in a reduction in perivascular polarization along the penetrating arteries, were associated with a significant decrease in the efficacy of the glymphatic flow. Under conditions of decreased polarization, the clearance of injected Aβ was significantly impaired in elderly mice, with a reduction of 40% compared to that in young mice [111]. Interestingly, the correlation between glymphatic system activity and Aβ deposition appears to be bidirectional. It has been shown that soluble AβOs can cause the delocalization of AQP4 from the end-feet to the soma of the astrocytes, leading to glymphatic dysfunction [95]. Importantly, this study also showed that glymphatic transport is suppressed prior to the significant accumulation of Aβ, and even before the occurrence of substantial Aβ deposits, glymphatic function may be inhibited as a result of long-term exposure to Aβ40 in the subarachnoid CSF [95]. Interesting conclusions also come from a study on an animal model examining the connection between physical activity and glymphatic system function in aging mice. Voluntary wheel running leads to improved astrocytic AQP4 expression and polarization, which increases the efficiency of glymphatic clearance and thereby reduces Aβ peptide accumulation [114]. Physical exercise also results in a reduction in the number of activated astrocytes and microglia, leading to a decrease in neuroinflammation. Therefore, physical exercise has a positive effect on glymphatic activity and reduces the Aβ load, which translates into a better cognitive performance in animals [114]. Another common thread between aging and impaired glymphatic function is sleep disorders. The functioning of the glymphatic system drops dramatically during wakefulness, while the peak of its efficiency is achieved during sleep. During wakefulness, a 90% reduction in glymphatic clearance has been observed [90]. A study using PET imaging showed that even one night of sleep deprivation causes a significant increase in Aβ deposition in healthy individuals [115]. One explanation for this phenomenon is that during sleep, the level of noradrenaline decreases, which results in the expansion of the extracellular space of the brain, which in turn reduces the resistance to fluid flow [116]. This can increase the efficiency of glymphatic flow and clearance. The question arises as to how this correlates with the aging process and an increasing risk of AD. The aging process is associated with changes in sleep architecture, among other things; decreased sleep efficiency; and a decreased ability to maintain sleep, which results in an increased number of nocturnal awakenings and a decreased percentage of slow-wave sleep and rapid eye movement (REM) sleep time [117]. The effectiveness of glymphatic clearance is closely correlated with the prevalence of slow-wave activity [118]. What is particularly important is that the aging brain rarely enters stage 3 NREM, that is, deep sleep, spending more time in the superficial NREM stage [119]. This results in a drastic decline in the clearance of brain waste and may contribute to the occurrence of neurodegenerative diseases in the elderly [118,120]. An increased incidence of sleep disorders has been observed in the older population [121,122,123]. It is estimated that 40–70% of older adults have chronic sleep disorders, with 50% remaining undiagnosed [123]. Sleep disturbances characteristic of the normal aging process also appear to be closely related to AD [124]. Sleep disorders occur in 60–70% of people with dementia or cognitive disorders and are associated with a worse prognosis of the disease and a poorer quality of life [125]. Sleep disruptions have been shown to be associated with more severe cognitive impairments and an accelerated course of AD [124,126,127,128]. Moreover, the severity of sleep disorders increases with disease [129].

Extensive research has established that APOE-ε4 modulates the pathology of Aβ and tau protein, influencing their aggregation and clearance, as well as contributing to neuroinflammation [130]. Studies involving mouse models expressing APOE-ε4 have demonstrated the increased deposition of tau and Aβ proteins [131,132]. Furthermore, APOE-ε4 may impair the function of the blood–brain barrier (BBB), as well as altering cerebral vascular function, which can affect the glymphatic system [90,107,133]. Despite the current limitations in the data regarding the effects of APOE-ε4 on the glymphatic system, further research is essential to elucidate APOE-ε4’s role in glymphatic clearance.

A 2022 rodent study reported that removal of fluid and metabolites from the intraocular space also occurs via glymphatic clearance. Aβ is cleared from the retina and the vitreous via the AQP4-dependent pathway and is driven by the ocular–cranial pressure difference [134]. It is important to conclude that this pathway may be impaired in glaucoma [134]. What is particularly interesting is that a possible connection between glaucoma and AD was considered almost 20 years earlier. A 2002 study reported that among the AD population, the incidence of glaucoma was significantly higher (25.9%) than that in a control group (5.2%) [135]. One year later, a study was published that considered chronic amyloid-beta neurotoxicity in a manner that mimicked AD in the pathogenesis of glaucoma, suggesting the potential implication of AD-dedicated therapy in glaucoma, and vice versa [136]. Over the years, there has been a consensus that both of these diseases have many common features that may belong to one spectrum of disease, but it is emphasized that missing links in the relationship between these two disorders remain that require explanation [137]. Is the glymphatic system one of the missing links between both diseases and the potential target of therapy for both disorders? Thus, this issue is worth considering.

## 4. The Glymphatic System—A Novel Potential Therapeutic Target

**Figure 3:**
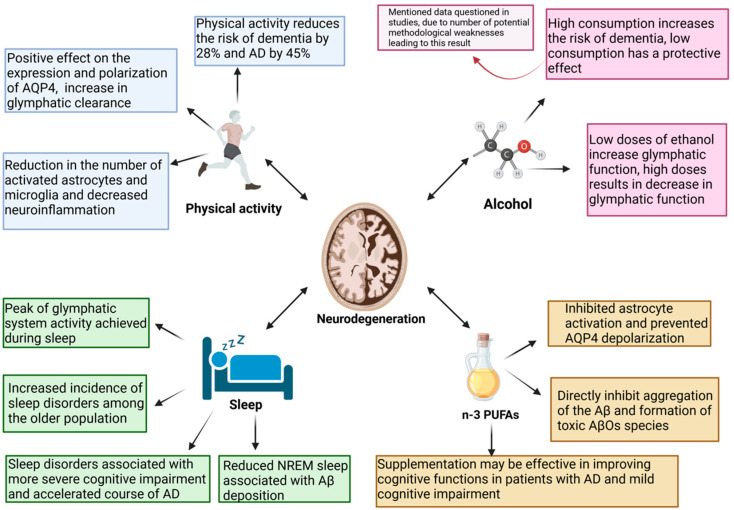
Potential factors influencing AD pathogenesis and glymphatic system activity. “Created in BioRender. Kopeć, K. (2025) https://BioRender.com/nz44w9n (accessed on 7 July 2025)”.

Influencing the glymphatic system to improve its function seems to have a potentially significant impact on the treatment of neurodegenerative disorders, including AD, considering how glymphatic clearance might be intertwined with many aspects of neurodegeneration pathogenesis. Lifestyle changes seem to have great potential to improve glymphatic system function [116,138]. Physical activity reduces the risk of dementia and AD by 28% and 45%, respectively [139]. The impact of physical activity on the function of patients with AD remains a subject of debate, but physical activity is mentioned as one of the main modulators of the risk of developing this disease [140]. The question arises as to whether this may be related to glymphatic activity. Animal studies have suggested this is likely. The above-mentioned study showed that voluntary exercise has a positive effect on the expression and polarization of AQP4, contributing to an increase in glymphatic clearance and promoting the removal of Aβ [114]. Hence, it is imperative to investigate the efficacy of aerobic exercise in individuals at various stages of AD in terms of AQP4 polarization and glymphatic flow, considering physical activity as a non-pharmacological approach to enhancing AD treatment [141]. Alcohol consumption is a modifiable risk factor for AD [142]. Research has suggested that ethanol has a J-shaped effect on the glymphatic system. Low doses of ethanol increase glymphatic function; however, chronic exposure to high doses of ethanol induces AQP4 mislocalization, leading to a dramatic decrease in glymphatic function [143]. Epidemiological data indicate that high alcohol consumption increases the risk of dementia, whereas low alcohol consumption has a protective effect, as shown by a meta-analysis [144,145]. This has been questioned in studies in which a number of potential weaknesses in the methodology that led to this result have been pointed out [146,147]. This issue requires clarification, and a broader examination of the glymphatic activity in the brain exposed to alcohol may provide interesting conclusions supporting or opposing controversial epidemiological data. Another important factor potentially influencing AD mentioned in studies discussing the impact of lifestyle on glymphatic system activity is the consumption of omega-3 polyunsaturated fatty acids (n-3 PUFAs) [116,138]. Supplementation with n-3 PUFAs in AD may be effective, especially in patients with very mild disease symptoms at its onset, thereby improving patients’ cognitive functions [148]. Furthermore, the effectiveness of n-3 PUFAs in improving cognitive functions in people with mild cognitive impairment has been demonstrated [149]. It has also been shown that the levels of n-3 are decreased in the brains of AD patients [150]. However, the results of several studies on the relationship between dietary n-3 PUFA intake and AD risk are inconsistent. A meta-analysis was performed to investigate the differences between study results [151]. The meta-analysis showed that an increase in fish intake of 100 g per week was associated with an 11% lower risk of AD. Nonetheless, it is noteworthy that in this study, there was no statistically significant correlation between n-3 PUFA intake and the reduction in AD risk [151]. However, a prospective study found that long-term n-3 PUFA supplementation protected cognition, reduced Aβ burden, and reduced the risk of AD in a population at genetic risk of *APOE* ε4 carriers [152]. Therefore, the effectiveness of term n-3 PUFA supplementation and determining the patient population that will benefit from either primary or secondary prevention remain topics for further research. It appears that their impact on AD can be attributed, among other factors, to their potentially beneficial effects on the glymphatic system. A study in an animal model showed that in mouse brains, after Aβ injection, n-3 PUFAs inhibited astrocyte activation and prevented AQP4 depolarization [153]. It is worth noting here that the beneficial effects of n-3 PUFAs expressed in the promotion of interstitial Aβ clearance from the brain were abolished in AQP4-knockout mice. Therefore, omega-3 polyunsaturated fatty acids facilitate the clearance of Aβ by regulating glymphatic function [153]. Furthermore, n-3 PUFAs directly inhibit the aggregation of Aβ and the formation of toxic AβO species, which interact bidirectionally with the glymphatic system [154]. The factors outlined above may potentially be associated with the pathogenesis of AD, exerting either protective or promoting effects on the progression of the disease and simultaneously being associated with the activity of the glymphatic system (Figure 3).

When discussing potential possibilities to enhance the functioning of the glymphatic system, it is imperative to acknowledge the significance of sleep and its pivotal role [155]. Sleep, which is crucial for the functioning of the glymphatic system, is necessary to maintain brain homeostasis and remove toxic metabolites that accumulate during periods of intense metabolism during wakefulness [156]. Due to their negative impact on glymphatic clearance, sleep disorders in AD are considered to perpetuate and potentially activate the pathomechanism of the disease [157]. Reduced NREM sleep is associated with Aβ deposition and tau accumulation in patients who are cognitively normal or show only mild impairment [158]. A recent study using a mouse model revealed that sleep can impact not only the clearance of Aβ but also its production [159]. These researchers found that fragmented sleep led to increased levels of Aβ in female mice, but this effect was not observed in males. This suggests a sex-dependent response to sleep fragmentation regarding Aβ regulation [159]. The heightened sensitivity of females to the increases in Aβ caused by disrupted sleep may help explain the higher incidence of AD found in women compared to men [159]. However, this remains a hypothesis that requires further investigation. Additionally, this study showed that the γ-secretase inhibitor Semagacestat was effective in blocking the rise in Aβ levels in female mice [159]. This study demonstrated that enhancing sleep quality in AD patients significantly lowers their blood levels of Aβ42/40 and Tau-pT181 biomarkers while also improving neuropsychological outcomes and reducing depression and anxiety symptoms [160]. Considering the bidirectional relationship between sleep and AD, an interesting direction of research will be to check whether improving sleep quality can delay the progression of the disease from preclinical to symptomatic or even reduce the risk of AD [161]. Due to the impact of sleep on the glymphatic system, it appears that therapeutic activities aimed at it may yield benefits for glymphatic clearance, potentially facilitating the treatment or prevention of neurodegenerative diseases. However, no studies have examined this issue [157]. Nonetheless, both pharmacological and non-pharmacological efforts are being made to address sleep disorders in AD patients. Currently, the available pharmacological therapies, such as antipsychotics and benzodiazepines, have an unfavorable side effect profile and are also expensive, which is why they are mainly used ad hoc [162]. Non-pharmacological approaches are currently preferred as the initial interventions. If these methods are unsuccessful, medications such as trazodone; melatonin; Z-drugs, including zopiclone and zolpidem; and relatively novel drugs such as dual orexin receptor antagonists are also used [163]. Several non-pharmacological therapies have also been shown to be beneficial. The potential direction of enhancing sleep in individuals with AD has been demonstrated through the utilization of Cognitive Behavioral Therapy for Insomnia (CBT-I), Bright Light Therapy, and Continuous Positive Airway Pressure (CPAP) treatment in patients with co-occurring sleep apnea [162]. Further research is needed in the field of therapy for sleep disorders in AD and into the role of the glymphatic system in this area. It is noteworthy that the selection of a highly effective and durable therapy for sleep disorders has not yet been feasible. This may represent a significant milestone in enhancing glymphatic function and provide fresh insights into the treatment of AD.

Another recently proposed therapeutic intervention that may have a beneficial effect on glymphatic system activity is hybrid electro-optical stimulation [164]. A study on an animal model of ischemic brain injury demonstrated that hybrid stimulation, combining electrical and optical stimulation, enhances neurological, motor, and cognitive functions and reduces brain atrophy following ischemia [164]. Importantly, this hybrid stimulation positively influences glymphatic clearance by modulating the polarization of aquaporin-4 (AQP4) channels. Enhanced glymphatic function may in turn contribute to a reduction in pro-inflammatory cytokine levels [164]. A potential therapeutic option being explored is also photobiomodulation (PBM) therapy [165,166]. PBM may have a beneficial effect on the functioning of the glymphatic system [165,166]. Preclinical studies in animal models have demonstrated that PBM reduces Aβ accumulation in the brain and enhances cognitive performance in AD mice [167]. Importantly, the therapeutic effects of PBM appear to be stronger during sleep compared to those in wakefulness [167]. However, it is important to note that most of this data has been derived from animal studies, and further research is necessary to confirm the effectiveness of PBM in humans [165,166].

## 5. Discussion

AD is one of the greatest challenges in modern neurology. The investigation of its pathogenesis has been ongoing for over a century [168]. Despite significant financial investments and years of research, the development of drugs has yet to yield satisfactory outcomes. The amyloid cascade hypothesis is the basis for numerous potential therapies being investigated [18]. The mechanism of action of aducanumab, a monoclonal antibody selective towards Aβ aggregates, is based on the premise of this cascade, wherein the primary causative factor of the pathogenetic process is the accumulation of Aβ aggregates in the brain. Despite the FDA’s registration of this molecule, the controversy surrounding it persists. Its effectiveness in enhancing cognitive functions has been questioned [28,29]. The question arises as to whether amyloid is actually the primary factor in the pathogenesis of AD. Although there is a discernible tendency towards deviating from the presumptions of the amyloid cascade hypothesis, the significance of Aβ cannot be completely excluded. Therefore, it is worth considering neurotoxic AβOs [45,46,47,48]. They are toxic through numerous mechanisms. One mechanism for the direct neurotoxicity of AβOs is the formation of voltage-independent, non-selective ion channel pores in the neuronal membranes [49]. This leads to the disruption of intracellular Ca^2+^ homeostasis, which in turn contributes to neurodegeneration [50]. The disruption of calcium homeostasis is associated with impairments in glutamatergic synaptic transmission [53]. Glutamate is one of the key neurotransmitters; however, in pathological conditions, it acquires neurotoxic functions, among others, by activating the NMDA receptors, which results in Ca^2+^-dependent cell death [55]. Furthermore, it has been demonstrated that AβOs induce mitochondrial oxidative stress and disrupt mitochondrial transport and integrity [60,61]. Neuroinflammation is a fundamental component of AD pathogenesis, and AβOs play a significant role in this process by inducing a pro-inflammatory phenotype in the microglia [68,69]. Furthermore, they interact directly with the NLRP3 inflammasome and release pro-inflammatory cytokines [73]. The rationale behind this modification to the notion of the significance of specific forms of Aβ in the pathogenesis of AD is also substantiated by accumulating evidence indicating the significance of the glymphatic system in AD. The study that coined the term glymphatic system reported that fluorescent-tagged soluble Aβ1–40 injected into the brain parenchyma was removed from the brain via the glymphatic pathway [85]. It is important that glymphatic clearance and amyloid pathology are interdependent. In the vicious circle of AD pathogenesis, impairment of the glymphatic system increases the accumulation of Aβ in the brain, and Aβ itself negatively affects glymphatic clearance [95,100,107]. It has been shown that soluble AβOs disrupt the polarization of AQP4, leading to glymphatic dysfunction, and importantly, glymphatic transport is suppressed prior to significant accumulation of Aβ [95]. The efficiency of the glymphatic system decreases with increasing age. One of the causes of this is disordered AQP4 polarization in aging brains, which correlates both with a decrease in Aβ clearance and an increasing incidence of AD in the elderly [111,113]. The aging process is associated with changes in sleep architecture, which is crucial for glymphatic clearance [90,117]. Moreover, sleep disorders, observed with an increased frequency in the AD population, are related to more severe cognitive impairments and an accelerated course of AD [124,126,127]. Sleep disorders are associated with a worse prognosis and a lower quality of life for patients [125]. The importance of sleep in glymphatic activity makes sleep disorders potential therapeutic targets. Early treatment of sleep disorders prior to the onset of the clinical symptoms of the disease and a diagnosis of AD may have the potential to delay the onset of symptoms and potentially alleviate their course. Nonetheless, to address this question, it is imperative to conduct further research to verify this hypothesis. Physical activity also has a beneficial effect on glymphatic clearance. In aging mice, wheel running was associated with improved expression and polarization of AQP4 and an increase in the efficiency of glymphatic clearance, which was reflected in a reduction in Aβ accumulation [114]. Moreover, physical activity was shown to be associated with a reduction in neuroinflammation, a well-known driver of neurodegeneration [114]. Lifestyle changes are known modulators of AD risk in humans [116]. Engaging in physical activity has been shown to significantly reduce the risk of dementia by 28% and AD by 45% [139]. Studying the glymphatic system activity in individuals engaged in a specific form of exercise may yield insights into the optimal physical activity for improving the efficacy of glymphatic clearance. Alcohol consumption is a modifiable factor in AD development related to the glymphatic system. One study demonstrated the impact of ethanol on the glymphatic system, indicating that low doses of ethanol enhanced glymphatic function, whereas chronic exposure to high doses of ethanol resulted in a significant decrease in glymphatic function [143]. This appears to be consistent with the results of a meta-analysis that showed that high alcohol consumption increased the risk of dementia, while low alcohol consumption had a protective effect [144,145]. However, these results have been questioned because of methodological weaknesses [146,147]. Conclusively determining the impact of alcohol on both the glymphatic system and the risk of dementia requires further research. It is also noteworthy that n-3 PUFAs have also been consumed. According to the results of recent studies, long-term n-3 PUFA supplementation reduced the Aβ burden and the risk of AD in a population at genetic risk of APOE ε4 carriers [152]. Reports have also indicated the efficacy of n-3 PUFA supplementation in the initial stages of AD and its enhancement of the cognitive abilities of individuals with mild cognitive impairments [148,149]. Research indicates that n-3 PUFAs exert a multidirectional impact on the glymphatic system, including the inhibition of astrocyte activation and the prevention of AQP4 depolarization [153]. Additionally, they inhibit the aggregation of Aβ and the formation of toxic AβOs [154]. The glymphatic system plays a significant role in AD pathogenesis. Whether it is important for effective therapeutic interventions to increase its efficiency to slow down or inhibit the development of the disease requires further clarification. Further justification for the modifiable AD risk factors discussed in the text can be found in their impact on the glymphatic system. Therefore, the prevention of AD is associated with ensuring the optimal functioning of glymphatic clearance. In the context of new therapies, the importance of the glymphatic system in AD requires further research. Can therapeutic activities aimed at the glymphatic system undertaken after the onset of clinical symptoms, which equates to a long-term process of pathological changes, benefit patients? This inquiry remains unresolved, yet it is undoubtedly a compelling and promising avenue for research in this domain.

## Data Availability

No new data were created or analyzed in this study. Data sharing was not applicable in this study.
